# Elite athletes’ overall oral health, values and related quality of life: a cross-sectional study

**DOI:** 10.1038/s41598-025-10479-z

**Published:** 2025-07-15

**Authors:** André Júdice, Diogo Brandão, João Botelho, Vanessa Machado, Luís Proença, Luciano M. A. Ferreira, Athanasios Stamos, Peter Fine, José João Mendes

**Affiliations:** 1 Egas Moniz Center for Interdisciplinary Research (CiiEM), Egas Moniz School of Health & Science, 2829-511 Almada, Portugal; 2 European Association for Sports Dentistry (EA4SD), Rambouillet, France; 3 UCL Eastman Dental Institute, London, UK

**Keywords:** Sports dentistry, Dental caries, Periodontal disease, Dental wear, Epidemiology, Oral health, Health care, Diseases, Dental diseases, Oral diseases

## Abstract

The present observational study aimed to identify the prevalence of oral health issues in this population and explore the relationship between oral health and factors relevant to athletic performance. One hundred and fourteen elite athletes triaged at a Sports Dentistry department from a university clinic were included for oral health assessment, self-reported periodontitis, basic erosive wear examination, and dental caries experience. They answered a sociodemographic questionnaire, oral health impact profile 14 to measure OHRQoL and OHV scale. Descriptive and inferential statistics assessed oral health outcomes and demographic or sport-related factors. Multivariable logistic regression explored predictors of poor oral health outcomes. The results reveal a high prevalence of oral health issues among elite athletes, with over half of the participants (51.8%) presenting two or more pathological or functional findings. Periodontal disease (55.1%), particularly gingivitis (51.8%), and dental caries (47.4%) were the two most common conditions, affecting nearly half of athletes. This high prevalence of oral conditions did not associate with worse OHRQoL and OHV. These results suggest that elite athletes face unique challenges regarding oral health, with some sex differences in oral health behaviors. Despite the high prevalence of oral diseases, related quality of life and values did not show any association, possibly due to the low perception of athletes.

**Supplementary Information:**

The online version contains supplementary material available at 10.1038/s41598-025-10479-z.

## Introduction

Oral health is an essential component of overall health and well-being^1,2^, influencing quality of life, daily functioning, and well-being which indirectly may influence performance^3^. Oral health has been considered as an essential component of Quality of Life (QoL) but has proved to be elusive when trying to measure its impact^4^. Spanemberg et al. suggested four categories indicating how oral health of adult individuals affects their QoL: ‘i) functional factors, ii) psychological factors, iii) social factors and iv) existence of discomfort or pain’^4^.

Elite athletes are typically defined as individuals who compete at the highest levels of their sport, such as national or international competitions, and who engage in rigorous, specialized training regimens to optimize performance, although there are a variety of definitions^5^. Unlike recreational athletes, elite competitors face intense physical, psychological, and biomechanical demands, often pushing their bodies to the limits of endurance and resilience. These high-performance requirements, combined with repetitive strain, increased stress levels, and the need for peak musculoskeletal function, can contribute to a heightened risk of temporomandibular joint (TMJ) disorders, facial pain, and related conditions.

Among elite athletes, oral health holds particular importance, as poor oral health has been linked to decreased athletic performance, increased injury risk, and systemic health issues^6,7^. Despite its significance, oral health in elite sports populations remains an often-overlooked aspect of sports medicine. To address this gap in knowledge, standardized assessment tools have been developed. The universal protocol for dental examination in sports offers a consistent approach to evaluating oral health across studies^8^. Furthermore, subjective measures such as Oral Health-Related Quality of Life (OHRQoL), through Oral Health Impact Profile-14 (OHIP-14), and Oral Health Value Scale (OHVS) provide valuable insights into the personal and professional ramifications of oral health for athletes^8^. These tools collectively form a comprehensive framework for assessing the oral health status of elite athletes^5^.

In this study, a cohort of elite athletes’ overall oral health using the universal protocol for dental examination in sports, OHIP-14 and OHVS questionnaires, was assessed. This investigation aimed to identify the prevalence of oral health issues in this population and explore the relationship between oral health and factors relevant to athletic performance.

## Materials and methods

### Study design and setting

This cross-sectional study evaluated the oral health of elite athletes using a universal protocol for dental examination in sports, employing the STROBE (Strengthening the Reporting of Observational Studies in Epidemiology) statement^9^ to ensure clarity and transparency.

The research was conducted at the Sports Dentistry department of a university clinic (Egas Moniz Dental Clinic, Almada, Portugal) and approved by the Review Institutional Board (Ethics Committee ID nº. 1101). This research has been performed in accordance with the Declaration of Helsinki. Written informed consent was obtained from all participants. Data collection occurred between July 2023 and November 2024 encompassing a fourteen-month period. Thus, the final sample size was based on a time-predefined sampling procedure.

### Participants and study size

Eligible participants included elite athletes aged 18 years old or older; who were actively training or competing during the study period; and who accepted to participate in the study. Participants were recruited through direct invitations. Exclusion criteria included recent dental treatments or inability to complete the required assessments. We did not have paralympic athletes participating in the study.

### Variables

The primary outcome was the overall oral health status assessed using the recently proposed universal protocol for dental examination in sports created by the European Association for Sports Dentistry (EA4SD) / Academy for Sports Dentistry (ASD)^8^.

As secondary outcomes OHRQoL was included, through OHIP-14, OHVS, sociodemographic characteristics and behavioral aspects, training intensity, and sport-specific factors.

### Data sources and measurements

The EA4SD/ASD protocol is an extensive tool that includes mandatory fields including demographic details, dental history and pain history^8^. All mandatory fields in all sections except for Sect. 6 due to the lack of paralympic athletes in the sample, were completed. Two modifications to the suggested protocol were undertaken: in the teeth examination, we performed the ICDAS instead of DMF index due to the lack of cutoff points in DMF for categorizing as ‘pathological or functional findings’; in the periodontal screening, we performed a validated self-reported periodontitis approach with 13 questions (Machado et al. 2021) instead of periodontal pocket depth, yet we maintained the mobility and gingival index.

The Basic Erosive Wear Examination (BEWE) was implemented^10^, to assess erosive lesions on all permanent teeth surfaces, except for third molars. In each sextant, the most affected surface was documented, and the sum of these scores was computed. The total was then utilized to assign an individual risk level as follows: no risk (BEWE ≤ 2); mild risk (3 < BEWE < 8); moderate (9 < BEWE < 13); high (BEWE ≥ 14)^10^.

Caries prevalence was assessed using the International caries detection and assessment system II (ICDAS II)^11^ considering its higher sensitivity in estimating caries prevalence compared to other criteria^12^. As per ICDAS criteria, sites were recorded by a 0 to 6 scoring system: 0 = sound; 1 = first visual change in enamel; 2 = distinct visual change in enamel; 3 = localized enamel breakdown (without clinical visual signs of dentinal involvement); 4 = Underlying dark shadow from dentin; 5 = Distinct cavity with visible dentin; 6 = Extensive distinct cavity with visible dentin. Patients with carious lesions received required treatment.

#### Sociodemographic questionnaire

Data were collected through a self-reported questionnaire on sociodemographic characteristics and behavioral aspects. This questionnaire was administered prior to taking panoramic radiographic and clinical oral observation. Overall, the information collected included demographic data: gender, age, marital status (categorized as single, married/cohabiting, divorced or widowed), level of education (categorized as elementary, middle, and higher education), occupation (categorized as student, employed, unemployed or retired), medical conditions. Smoking habits was categorized according to the National Health and Nutrition Examination Survey (NHANES) methodology as non-smoker (never smoked or smoked less than 100 cigarettes in life), ex-smoker (smoked less at least 100 cigarettes in life and currently does not smoke) and active smoker (smoked less at least 100 cigarettes in life and currently smokes)^13^. The number of questioned how many cigarettes smoked per day and for how long was also enquired about. Alcohol consumption was dichotomized as ‘yes’ or ‘no’.

#### Oral health values scale (OHVS)

To measure the priority and relevance of oral healthcare for each participant, the Portuguese-validated version of the OHVS was used^14,15^. The Oral Health Value Scale (OHVS) is a comprehensive assessment tool that evaluates multiple domains of oral health to comprehensively understand a patient’s oral health status^14^, This instrument consists of 12 items that cover professional dental care (items 4, 8 and 11), appearance and health (items 3, 7 and 12), flossing (items 2, 5 and 10), and retaining natural teeth (items 1, 6 and 9). Each question is rated on a 5-point Likert scale^16^with responses ranging from ‘strongly disagree’ to ‘strongly agree’, allowing for a nuanced understanding of the patient’s responses and enabling the dental professional to identify and address any potential issues in a timely manner.

#### Oral health-related quality of life (OHRQoL)

The Portuguese-validated version of the Oral Health Impact Profile-14 (OHIP-14) was used to evaluate the impact of oral health on the quality of life of each study participant^17^. The OHIP-14 consists of 14 questions that are used to appraise to which extent oral health problems interfere with various aspects of a patient’s life, including physical pain, physical disability, psychological discomfort, and social discomfort^18^. The questionnaire discloses how often the individual experienced such a situation is rated on a 5-point scale for each question (never, hardly ever, occasionally, often, very often)^18^.

#### Gingivitis and self-reported measures of periodontitis

A case was classified as gingivitis if the participant did not self-report periodontitis but exhibited gingivitis according to the Loe & Silness approach^19^.

The screening of periodontal status was carried out using a self-reporting approach previously validated using thirteen questions, of which two have an area under the curve of 0.8 of predictive ability the number of teeth lost observed clinically^20^. The remaining questions included information regarding gum and teeth health, loose teeth, bone loss, tooth appearance, and the use of dental floss and mouthwash.

### Quantitative variables

Data were categorized as follows: ICDAS scores were dichotomized as ‘yes’ or ‘no’ (if receiving a score of 3 or higher a ‘yes’ was attributed). OHIP-14 and OHVS scores were analyzed as continuous variables. Self-reported periodontal health was categorized as ‘yes’ or ‘no’.

### Statistical analysis

Descriptive statistics (mean, standard deviation (SD), frequencies and percentages) were calculated for all variables, according to its type. Considering the importance of reporting data according to sex, we decided to analyze data separately for men and women, by using inferential comparative statistical procedures (Student’s t-test, ANOVA). Additionally bivariate analyses (Chi-square test) were used to identify associations between oral health outcomes and demographic or sport-related factors. Multivariable logistic regression models were employed to adjust for potential confounders and assess predictors of poor oral health outcomes. Statistical significance was set at 5% (p < = 0.05). All analyses were conducted using R.

## Results

### Participants and characteristics

**Fig. 1:**
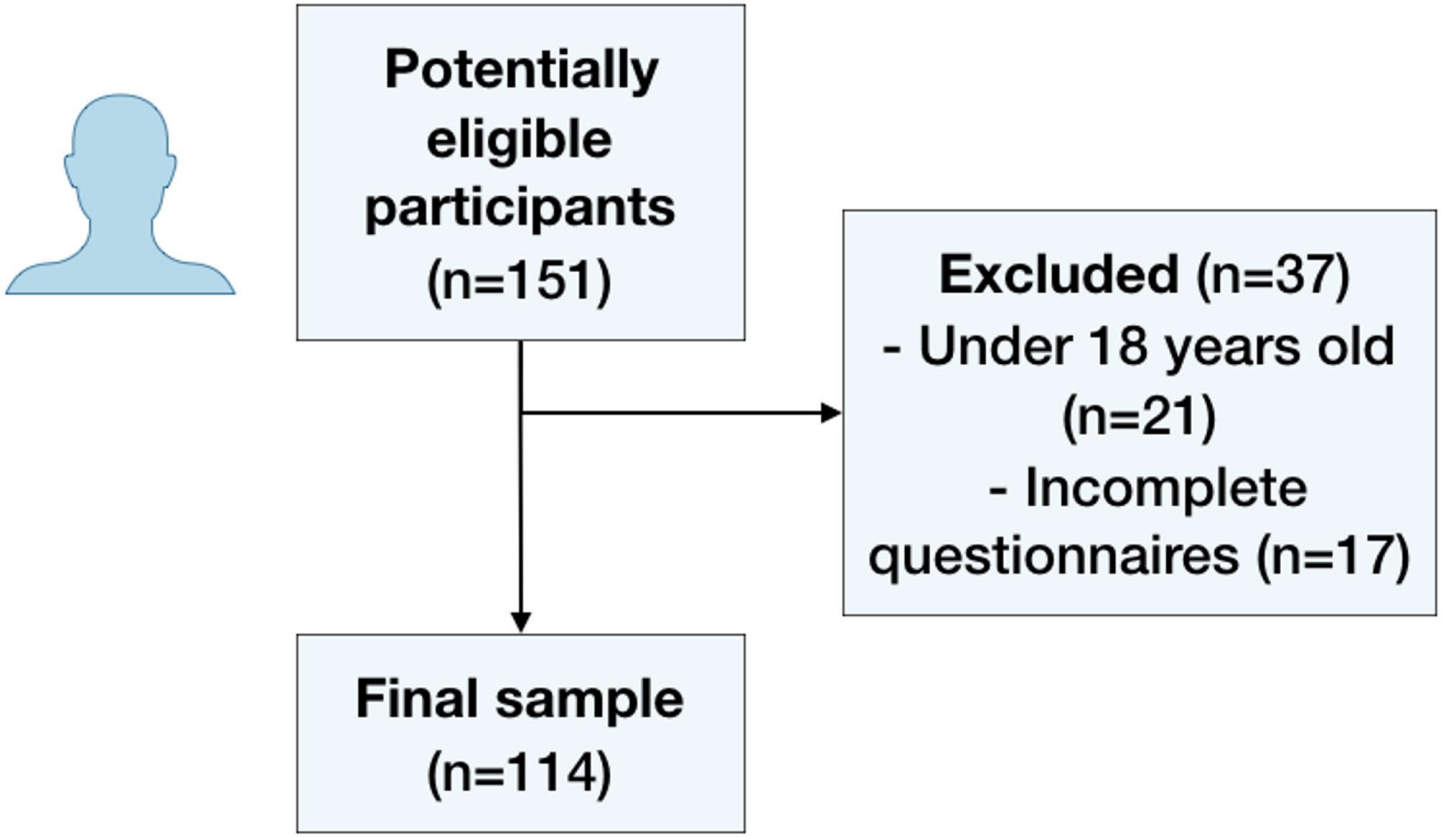
Flowchart of participants.

From an initial sample of 151 elite athletes, 21 were excluded for being minors, and 16 were excluded due to incomplete questionnaires, resulting in a final sample of 114 participants (Fig. 1). Overall, this cohort was mainly male (68.4%, *n* = 78), with an average age of 25.4 (± 5.2) years. Most athletes were practicing; football (80.7%, *n* = 92), basketball (7.0, *n* = 8) or martial arts (4.4, *n* = 5) (Table 1). Only 7.9% (*n* = 9) of the athletes had no pathological or functional findings, while 51.8% (*n* = 59) had two or more conditions that may compromise eligibility to practice sports.

There were no statistically significant differences in age between males and females (*p* = 0.113). Regarding oral health behaviors, males reported significantly higher consumption of sugary drinks, lollipops, and sports drinks compared to females (*p* = 0.007), while females demonstrated significantly greater use of mouthguards (*p* = 0.012). No significant differences between sex were observed in the timing of the last dental visit (*p* = 0.408), dental checkup frequency (*p* = 0.122), OHIP-14 scores (*p* = 0.772) or OHVS (*p* = 0.278).

**Table 1:** Participants characteristics.

	Total (*N* = 114)	Male (*n* = 78)	Female (*n* = 36)	*p*-value
Age, mean (SD)	25.4 (5.2)	25.9 (5.3)	24.4 (4.7)	0.113
EA4SD/ASD protocol outcome				
Green (no pathological or functional findings)	7.9 (9)	6.4 (5)	11.1 (4)	0.688
Yellow (one pathological or functional finding)	40.4 (46)	41.0 (32)	38.9 (14)	
Red (≥ 2 pathological or functional findings)	51.8 (59)	52.6 (41)	50.0 (18)	
Last dental visit				
≤ 6 months	11.4 (13)	14.1 (11)	5.6 (2)	0.408
6–12 months	57.9 (66)	56.4 (44)	61.1 (22)	
> 12 months	30.7 (35)	29.5 (23)	33.3 (12)	
Dental checkups frequency				
< 1 year	25.4 (29)	26.9 (21)	22.2 (8)	0.122
1/year	64.0 (73)	59.0 (46)	75.0 (27)	
2 or more/year	10.5 (12)	14.1 (11)	2.8 (1)	
Experienced jaw injury	4.4 (5)	3.8 (3)	5.6 (2)	0.104
Sodas, lollipops, sports drinks (number per day)	1.4 (1.2)	1.6 (1.4)	1.1 (0.6)	**0.007**
Smoking/chew tobacco (times per day)	0 (0)	0 (0)	0 (0)	–
Alcohol drinks per day	0 (0)	0 (0)	0 (0)	–
Mouthguards	12.3 (14)	6.4 (5)	25.0 (9)	**0.012**
OHIP-14	8.1 (11.7)	7.9 (11.3)	8.6 (12.6)	0.772
OHVS (%), mean (SD)	54.2 (9.1)	54.8 (9.7)	52.9 (7.8)	0.278

*OHIP-14* oral health impact profile-14, *OHVS* oral health value scale.

p-values < = 0.05 denoted in bold.

### Outcome data

According to the EA4SD/ASD protocol outcomes, most participants (51.8%) presented with two or more pathological or functional findings (red category), with no significant differences between sexes (*p* = 0.688). We found significant differences in the average age of athletes with green EA4SD/ASD protocol outcome, OHVS and OHRQoL (*p* < 0.001) (Table 2).

**Table 2:** Participants distribution according to the EA4SD/ASD protocol outcomes.

	EA4SD/ASD protocol outcome			
	Green (*n* = 9)	Yellow (*n* = 46)	Red (*n* = 59)	p-value
Age, mean (SD)	28.3 (7.30)	24.6 (4.8)	25.7 (5.0)	< 0.001
OHIP-14, mean (SD)	3.9 (4.6)	8.3 (14.7)	8.7 (9.6)	< 0.001
OHVS (%), mean (SD)	50.7 (4.0)	54.9 (10.1)	54.1 (8.9)	< 0.001

*OHIP-14* oral health impact profile-14, *OHVS* oral health value scale.

### Prevalence of oral health issues

Periodontal disease was the most prevalent condition diagnosed (55.1%) with a predominance of gingivitis, with dental caries being the second (*n* = 54, 47.4%) (Table 3). We observed similar prevalence of erosion risk (BEWE ≥ 2) among males (38.5%) and females (27.8%) (*p* = 0.3682p = 0.3682p = 0.3682) and dental caries (ICDAS ≥ 3) in 47.4% of participants overall, with males (44.9%) and females (52.8%) showing no significant differences (*p* = 0.726). Gingivitis was the most common oral condition (51.8%), with slightly higher prevalence among females (55.6%) compared to males (50.0%) (*p* = 0.208). Periodontitis, although uncommon, was exclusively found in males (7.7%) and not in females (*p* = 0.167).

Regarding temporomandibular joint (TMJ) and facial pain, 35.1% of participants reported pain in the face and/or temple, with similar proportions between men and women (*p* = 0.559). TMJ noise was reported by 9.6% of participants, more frequently in females (16.7%) than males (6.4%), though not statistically significant (*p* = 0.461). Pain on TMJ palpation was significantly more common among women (22.2% vs. 3.8%, *p* = 0.0060). Similarly, headaches were significantly more prevalent in women (16.7%) than males (3.8%) (*p* = 0.047). Pain on muscle palpation was reported by 35.1% of participants, with a higher prevalence in women (44.4%) compared to men (30.8%) (*p* = 0.226), although not statistically significant. No participants exhibited mandibular movement limitations.

**Table 3:** Participants characteristics.

	Total (*n* = 114)	Male (*n* = 78)	Female (*n* = 36)	*p*-value
EA4SD/ASD protocol outcome				
Green (no pathological or functional findings)	7.9 (9)	6.4 (5)	11.1 (4)	0.688
Yellow (one pathological or functional finding)	40.4 (46)	41.0 (32)	38.9 (14)	
Red (≥ 2 pathological or functional findings)	51.8 (59)	52.6 (41)	50.0 (18)	
Erosion risk (BEWE ≥ 2)	35.1 (40)	38.5 (30)	27.8 (10)	0.368
Dental caries (ICDAS ≥ 3)	47.4 (54)	44.9 (35)	52.8 (19)	0.726
Periodontal diseases	55.1 (65)	57.7 (45)	55.6 (20)	0.187
Gingivitis	51.8 (59)	50.0 (39)	55.6 (20)	0.208
Periodontitis	5.3 (6)	7.7 (6)	0 (0)	0.167
Reported pain in the face and/or temple	35.1 (40)	38.5 (30)	27.8 (10)	0.559
TMJ noise	9.6 (11)	6.4 (5)	16.7 (6)	0.461
Mandibular movement limitations	0 (0)	0 (0)	0 (0)	-
Pain on muscle palpation	35.1 (40)	30.8 (24)	44.4 (16)	0.226
Pain on TMJ palpation	9.6 (11)	3.8 (3)	22.2 (8)	**0.006**
Headaches present	7.9 (9)	3.8 (3)	16.7 (6)	**0.047**

p-values < = 0.05 denoted in bold.

### Relationship between oral health status with OHRQoL and OHVS

When using a multivariable logistic regression analysis, while exploring factors associated with achieving a green EA4SD/ASD protocol outcome (Table 4), age was found to be significantly associated with the referred outcome (OR: 1.25, 95% CI: 1.04–1.62, *p* = 0.033). Conversely, the OHIP-14 score, albeit demonstrating a high odds ratio value (OR: 8.64, 95% CI: 0.50–214.48), was non-significant (*p* = 0.149). Similarly, OHVS percentage showed no significant contribution (OR: 1.52, 95% CI: 0.00-2.88, *p* = 0.888).

**Table 4:** Multivariable logistic regression analysis towards a green EA4SD/ASD protocol outcome.

	OR	OR (95% CI)	*p*-value
Age, mean (SD)	1.25	1.04–1.62	0.033
OHIP-14 score, mean (SD)	8.64	0.50-214.48	0.149
OHVS (%), mean (SD)	1.52	0.00-2.88	0.888

*OR* odds ratio.

## Discussion

This study assessed the oral health status of elite athletes, utilizing the universal protocol for dental examination in sports, the OHIP-14 for oral health-related quality of life (OHRQoL), and the OHV scale for oral health values. The results reveal a high prevalence of oral health issues among elite athletes, with over half of the participants (51.8%) presenting two or more pathological or functional findings. Periodontal disease (55.1%), particularly gingivitis (51.8%), was the most prevalent condition, affecting over half of athletes, followed by dental caries (47.4%). These findings align with previous clinical studies that reported data for dental caries, tooth erosion, gingivitis and periodontitis^7,21–31^. Previous studies have been reporting data from a variety of sports (soccer, rugby, swimmers, biathletes, or multiple sports), ages (from young to adult athletes), or professional activities (from non-professional, professional or elite athletes). From a methodological standpoint, our study closely resembles Gallagher et al.^23^ elite and professional athletes and Kragt et al.^25^ on pre-Olympic oral state assessment, with results being very similar to the latter. When considering the very high percentage of football players in our cohort, our study outcomes also find parallels to those presented by Needleman et al.^21^ (with players having 37% active dental caries, 53% dental erosion and 5% moderate-severe irreversible periodontal disease) and Solleveld et al.^22^ (with 34% of players self-reporting gum problems). Collectively, these results suggest that elite athletes face unique challenges to maintaining oral health, including dietary habits, stress, and demanding training schedules. Notably, this study also highlights differences in oral health behaviors and outcomes by gender, with males reporting higher consumption of sugary beverages and females demonstrating greater use of protective measures such as mouthguards.

Oral health issues can negatively impact athletic performance through pain, systemic inflammation, and psychological discomfort^25^. The high prevalence of gingivitis and dental caries observed in this study underscores the need for targeted interventions to improve oral health in this population. Additionally, the significant association between age and better oral health outcomes (green EA4SD/ASD protocol outcome) suggests that more experienced athletes may adopt better oral health practices, potentially due to greater awareness or access to care. However, no significant associations were found between OHRQoL or OHV scores and the EA4SD/ASD protocol outcomes, indicating that these subjective measures may not directly correlate with clinical findings in this cohort.

Although the overall prevalence of oral health conditions did not vary significantly between sexes, certain behaviors and symptoms did. Females reported a notably higher use of mouthguards, possibly due to greater awareness of the risk of oro-facial trauma in sports or differences in sport-specific requirements. In contrast, males showed a higher consumption of sugary drinks, which could increase their risk for dental caries and erosion. These findings underscore the importance of tailoring oral health interventions to address gender-specific behaviors and needs.

Temporomandibular joint (TMJ) and facial pain were reported by a substantial proportion of athletes in this study, with significantly higher prevalence of pain on TMJ palpation and headaches among females. These findings are consistent with previous studies indicating that TMJ disorders and facial pain are more common in females, possibly due to hormonal, anatomical, or behavioral factors^32,33^. Given this heightened susceptibility, increasing awareness and proactive screening in female athletes could facilitate early intervention, potentially reducing the impact on performance and overall well-being. Addressing these conditions is essential, as they can affect not only oral health but also overall quality of life and athletic performance.

Our findings highlight the importance of incorporating oral health into sports medicine protocols, as previously recommended by Needleman et al.^34^ To address the high prevalence of oral health issues among elite athletes, regular dental screenings, customized education, and preventive strategies should be implemented. Additionally, encouraging protective behaviors, such as using mouthguards, undergoing regular oral health screenings, and reducing the consumption of sugary beverages, can help mitigate the risks associated with oral health conditions.

### Strengths and limitations

A key strength of this study is the comprehensive approach to assessing oral health using validated tools, including the EA4SD/ASD protocol, OHIP-14, and OHV scale. The use of a self-reported periodontal screening tool, validated in a Portuguese population, adds further relevance to the study. However, limitations that should be noted include: the cross-sectional design precludes causal inferences, and the convenience consecutive sample, mainly of football athletes, may limit generalizability to other populations of elite athletes. Future research should explore longitudinal outcomes and intervention strategies to improve oral health and its impact on athletic performance. Additionally, the absence of paralympic athletes in our cohort limits the scope of our findings, and future studies should aim to include this population to better understand their specific oral health challenges. We recommend that future research incorporates longitudinal multi-center and multi-sport studies to enhance the generalizability of results across diverse athlete populations. Furthermore, while self-reports present its advantages, they are susceptible to recall bias and subjective interpretation, which could affect the accuracy of predictions^35,36^. One might argue that athletes may under- or over-report oral health problems/ behaviors due to concerns about their image or performance, and this should be accounted for in the future. Other confounding variables, such as dietary habits, stress, and access to care, may influence both oral health and quality of life and were not possible to register in this study. Thus, capturing these potentially significant variables in upcoming studies may improve or overview on oral health related risk factors and indicators. As well, there is potential to increment the current universal protocol with other objective measures (e.g., salivary biomarkers, radiographs), that may improve the screening and diagnostic capacity in Sports Dentistry.

## Conclusion

This study provides important insights into the oral health status of elite athletes, emphasizing the prevalence of periodontal disease, dental caries, and TMJ-related issues. This high prevalence of oral conditions was not associated with worse OHRQoL and OHV. Future studies shall expand to multi-center and multi-sport studies to enhance the generalizability of results across diverse athlete populations.

## Electronic supplementary material

Below is the link to the electronic supplementary material.

Supplementary Material 1

Supplementary Material 2

## Data Availability

All data generated or analysed during this study are included in this published article as supplementary information file.
